# Testing strong factorial invariance using three-level structural equation modeling

**DOI:** 10.3389/fpsyg.2014.00745

**Published:** 2014-07-25

**Authors:** Suzanne Jak

**Affiliations:** Department of Methods and Statistics, Faculty of Social Sciences, Utrecht UniversityUtrecht, Netherlands

**Keywords:** measurement invariance, three-level structural equation modeling, cluster bias, measurement bias, multilevel SEM

## Abstract

Within structural equation modeling, the most prevalent model to investigate measurement bias is the multigroup model. Equal factor loadings and intercepts across groups in a multigroup model represent strong factorial invariance (absence of measurement bias) across groups. Although this approach is possible in principle, it is hardly practical when the number of groups is large or when the group size is relatively small. Jak et al. (2013) showed how strong factorial invariance across large numbers of groups can be tested in a multilevel structural equation modeling framework, by treating group as a random instead of a fixed variable. In the present study, this model is extended for use with three-level data. The proposed method is illustrated with an investigation of strong factorial invariance across 156 school classes and 50 schools in a Dutch dyscalculia test, using three-level structural equation modeling.

## Introduction

The purpose of this study is to show how three-level structural equation modeling (SEM) can be used to test for measurement invariance across the Level 2 and Level 3 clustering variables. The method is illustrated by testing measurement invariance across school classes and schools in a dyscalculia screening instrument.

### Measurement invariance

In order to meaningfully compare test scores across groups, the test should be measurement invariant with respect to group membership. When a test is measurement invariant, the differences in test scores across groups can be attributed to differences in the constructs that were intended to be measured. The importance of measurement invariance is widely recognized (Mellenbergh, 1989; Millsap and Everson, 1991; Meredith, 1993; Vandenberg and Lance, 2000). In order to establish whether a test is measurement invariant across groups, one should test the equality of measurement parameters across groups. With continuous normally distributed test scores and continuous normally distributed latent variables (factors), the linear factor model is the suitable measurement model (Mellenbergh, 1994). If the relation between the factors and the test scores are equivalent across studies (i.e., if factor loadings are equal across groups), weak factorial invariance (also labeled as metric invariance) holds. If in addition the intercepts are equivalent across groups, strong factorial invariance (also labeled as scalar invariance) holds. With strong factorial invariance, the means of the factors can be meaningfully compared across the groups. If in addition the residual variances are equivalent (strict factorial invariance), the observed means can be compared across groups (Meredith, 1993; Widaman and Reise, 1997). In this study I focus on strong factorial invariance.

### Strong factorial invariance across many groups

With a small number of groups, multigroup confirmatory factor analysis can be used to test the equality of measurement parameters (e.g., Wicherts and Dolan, 2010). If the number of groups is large, it may be convenient to view group as a random mode of variation, and use multilevel modeling (De Jong et al., 2007; Fox, 2010). See Muthén and Asparouhov (2013) for an overview of several fixed and random approaches to the study of measurement invariance across many groups.

Jak et al. (2013) showed how invariance restrictions across groups in a fixed model imply across level restrictions in a multilevel model. In a multilevel structural equation model, the covariance matrix is modeled as the sum of the covariance matrices at different levels (Muthén, 1990; Rabe-Hesketh et al., 2004). For a two-level model (for example, if the test scores are from students nested in school classes), the total covariance matrix can be decomposed in two independent covariance matrices:

(1) $$\Sigma_{\text{TOTAL}}=\Sigma_{\text{LEVEL2}}+\Sigma_{\text{LEVEL1}}.$$

The (pooled, within class) differences between students' scores are modeled by **Σ**_LEVEL1_. The average score of the school classes may also differ, these differences are modeled by **Σ**_LEVEL2_. At the different levels, distinct measurement models can be used to describe the covariances between the test scores. In this study we use linear factor models:

(2) $$\begin{array}{l} \Sigma_{\text{LEVEL2}}=\Lambda_{\text{LEVEL2}}\Phi_{\text{LEVEL2}}\Lambda_{\text{LEVEL2}}^{\text{t}}+\Theta_{\text{LEVEL2}},\\ \Sigma_{\text{LEVEL1}}\;=\Lambda_{\text{LEVEL1}}\Phi_{\text{LEVEL1}}\Lambda_{\text{LEVEL1}}^{\text{t}}+\Theta_{\text{LEVEL1}}. \end{array}$$

With *p* observed variables and *k* common factors, **Φ**_LEVEL2_ and **Φ**_LEVEL1_ are *k* by *k* covariance matrices of common factors, **Θ**_LEVEL2_ and **Θ**_LEVEL1_ are *p* by *p* (diagonal) matrices with residual variances, and **Λ**_LEVEL2_ and **Λ**_LEVEL1_ are *p* by *k* matrices with factor loadings at Level 2 and Level 1, respectively.

## Methods

### Strong factorial invariance in two-level models

As explained by Jak et al. (2013), with two-level data, strong factorial invariance across clusters implies:

$$\Sigma_{\text{LEVEL2}}\;=\Lambda \Phi_{\text{LEVEL2}}\Lambda^{\text{t}},$$

and

(3) $$\Sigma_{\text{LEVEL1}}\;=\Lambda \Phi_{\text{LEVEL1}}\Lambda^{\text{t}}+\Theta_{\text{LEVEL1}}.$$

This means that if there is strong factorial invariance across clusters (so the factor loadings and intercepts are equal across school classes), the factor loadings are equal across levels, and there is no residual variance at Level 2 (**Θ**_LEVEL2_ = **0**). All differences at the cluster (school class) level are thus differences in the common factor(s). If strong factorial invariance does not hold (i.e., if the intercepts differ across clusters), this results in residual variance at Level 2 (**Θ**_LEVEL2_ ≠ **0**). Strong factorial invariance across clusters can thus be investigated by testing the significance of Level 2 residual variance in a factor model with equal factor loadings across levels. This test is denoted the test for cluster bias. The cluster bias model can test whether strong invariance holds, but cannot differentiate between violations of weak and strong factorial invariance. The focus of this study is therefore on testing whether strong factorial invariance holds.

### Strong factorial invariance in three level models

With three-level data, such as test scores from students, nested in school classes, nested in schools, one may employ three-level structural equation modeling (Rabe-Hesketh et al., 2004). The total covariance matrix can be decomposed into three covariance matrices:

(4) $$\Sigma_{\text{TOTAL}}=\Sigma_{\text{LEVEL3}}+\Sigma_{\text{LEVEL2}}+\Sigma_{\text{LEVEL1}}.$$

Here, **Σ**_LEVEL3_ refers to the covariance matrix of school averages, **Σ**_LEVEL2_ refers to the covariance matrix of class deviations from the school average, and **Σ**_LEVEL1_ is a covariance matrix of students deviations from the class average.

In a three-level factor model, the common factors also exist (have variance) at the third level. For example, with data from children in school classes in schools, the school averages in the test scores may be different. If strong factorial invariance across schools and across school classes holds, then the following model holds:

$$\begin{array}{l} \Sigma_{\text{LEVEL3}}=\Lambda \Phi_{\text{LEVEL3}}\Lambda^{\text{t}},\\ \Sigma_{\text{LEVEL2}}=\Lambda \Phi_{\text{LEVEL2}}\Lambda^{\text{t}}, \end{array}$$

and

(5) $$\Sigma_{\text{LEVEL1}}=\Lambda \Phi_{\text{LEVEL1}}\Lambda^{\text{t}}+\Theta_{\text{LEVEL1}},$$

Where **Φ**_LEVEL3_ is a *k* by *k* covariance matrix of the common factors at Level 3. In this model, the common factor is the only source of variance at the class and at the school level (Rabe-Hesketh et al., 2004). If other variables than the common factor have influence at the school level, this will lead to residual variance at Level 3 (**Θ**_LEVEL3_ ≠ **0**), which means that measurement invariance across schools does not hold.

## Illustration

### Introduction

Testing measurement invariance across in three-level models will be illustrated by testing strong factorial invariance across school classes and across schools in a dyscalculia screening test. Developmental dyscalculia is a learning difficulty specific to mathematics learning (Butterworth, 2005; Devine et al., 2013). Children with developmental dyscalculia have deficits in understanding basic concepts such as quantity conservation and reversibility, despite otherwise typically developing mental abilities (Kosc, 1974; Gross-Tsur et al., 1996). Dyscalculia is estimated to affect between 1.3 and 10% of the population, which is equivalent to the prevalence of dyslexia (Devine et al.). The screening of dyscalculia will often take place in the school, where a teacher administers the test to all children in the class. This way, the teacher can have influence on the test scores of the children. For example, one teacher may give better instructions than the other, leading to better test scores (less findings of dyscalculia) in the last school class. If this happens, the test is not measurement invariant across school class, as differences in test scores are not fully attributable to differences in dyscalculia (but to differences in quality of the instruction). At the school level, the school system may have influence on the test scores. For example, one school may have a curriculum that involves a different method to teach mathematics than another school. Or some schools may use more paper and pencil tests than other schools, leading to more experience of the students with a testing situation than others. If this is the case, two students that are equal in their levels of dyscalculia, may score differently on a screening test, depending on the school they are in. It is therefore important to establish measurement invariance of an instrument across school classes and schools. In this example, strong factorial invariance of a Dutch screening instrument for dyscalculia is tested across school classes and schools.

### Methods

#### Data

Respondents were 4527 students from 156 school classes in 50 schools in the Netherlands, of which 20 secondary schools and 30 primary schools. In all schools, the parent-teacher association or the teacher gave permission for the administration of the test. The test was administered by the teacher during regular school time. The students were in the first grade of the secondary school, or in the last 3 years of primary school. The schools were located across the country in a way that is representative of the distribution of people living in The Netherlands. For some schools, the class identifier was missing, in which case we treated all observations to be in one cluster. The average number of respondents per class was 29.02, the average number of respondents in each school was 90.54. The mean age of the students was 11.42 (*SD* = 1.27), and 49.1% was a boy.

#### Instrument

The NDS (Nederlandse Dyscalculie Screener; Milikowski and Vermeire, 2013) is a screening instrument for dyscalculia. The screening instrument consists of eight subtests with a large set of items. For each subtest, the respondents try to answer as much items correctly as possible within 1 min. The score on each subtest is the amount of items answered correctly. The tests are long enough to ensure that no one can finish all questions in 1 min. See Appendix A for an overview of the content of the eight subtests. Before the respondents made the eight subtests they performed a control task, which does not involve numbers, to practice with the testing situation. The higher the score on each subtest, the lower the level of dyscalculia is assumed to be. As the scores are not recoded, the common factor that is assumed to underlie the test scores is actually the opposite of dyscalculia.

#### Analysis

All analyses were performed in the program M*plus* 7 (Muthén and Muthén, 1998–2012) using full information maximum likelihood estimation. In addition to the χ2 - statistic, the root mean squared error of approximation (RMSEA) and the comparative fit index (CFI) were used as measures of overall goodness-of-fit. RMSEA values smaller than 0.08 are satisfactory, values smaller than 0.05 indicate close fit (Browne and Cudeck, 1992). CFI values over 0.95 indicate reasonably good fit (Hu and Bentler, 1999).

First, the intraclass correlations and the significance of the variance at the class level and school level were inspected to decide whether multilevel modeling is actually necessary. Next, a measurement model is constructed at Level 1, with a saturated Level 2 and Level 3 model, so that all misfit stems from Level 1. Based on the final measurement model, a model with equal factor loadings across the three levels is fitted. Next, the significance of the Level 2 residual variance for all indicators is tested, by fixing all residual variance at Level 2 at zero. A significant chi-square difference in comparison with the free model indicates significant measurement bias across school classes. Finally, significance of Level 3 residual variance is tested by comparing the fit of a model with the residual variances at Level 3 fixed at zero with the model from the previous step. All tests are performed using a significance level of 5%.

Testing variances with the chi-square difference test in this way is not strictly correct Stoel et al. (2006). Correct testing requires the derivation of an asymptotic distribution of the likelihood ratio test statistic, which is a complex mixture of chi-square distributions. As this is beyond the scope of this work, I accept that the testing procedure is not correct, and keep in mind that it leads to an overly conservative test.

### Results

The intraclass correlations at the class level varied between 0.19 (Test 4) and 0.43 (Test 8), meaning that 19% to 48% of the variance in test scores is at the class level. At the school level the ICC's were much smaller, varying between 0.4% (Test 5) and 2% (Test 8). All variables showed significant variance at the class level, but not at the school level. Based on these results, one could decide to use two-level modeling instead of thee-level modeling. For the purpose of illustration, and because the interest is in differences between schools, I will continue the analyses using a three-level model.

First, the goal was to construct a measurement model at Level 1 with a saturated Level 2 and Level 3 model. Unfortunately, the model estimation did not converge when the Level 3 model was saturated, presumably because the saturated Level 3 model was overparameterized (i.e., some Level 3 correlations are actually zero). As a solution, the measurement model was specified with a saturated Level 2 model, and with corrections on the chi-square and standard errors to account for the dependency due to the school level (using “Type = Twolevel Complex” in M*plus*). A one-factor model fitted the data satisfactory according to the RMSEA, χ^2^_(20)_ = 304.51, *p* < 0.05, RMSEA = 0.056, CFI = 0.93. There was a modification index of a size 10 times larger than the others for the relation between Test 1 and Test 2. These tests are indeed quite similar (they both involve choosing the largest number, see Appendix A), so it seems to make sense that these tests share some specific variance. Adding a residual covariance between Test 1 and Test 2 leads to a better fitting model, χ^2^_(19)_ = 135.69, *p* < 0.05, RMSEA = 0.037, CFI = 0.97, with close fit according to the RMSEA and good fit based on the CFI. This model was accepted as the measurement model. Because it is not possible to model residual correlations at the higher levels in the next steps, the model was reparameterized by adding a factor on which Test 1 and Test 2 loaded. This factor was uncorrelated with the common factor, and both factor loadings are fixed at 1, so the model is equivalent with the model containing the correlated residuals (the estimate of the factor variance will be equal to the estimate of the residual covariance).

Using this measurement model, strong factorial invariance across school classes and schools is investigated. A model with equal factor loadings across levels fitted the data satisfactorily (see Model 1 in Table 1). Fixing the Level 2 residual variance at zero deteriorated the model fit significantly [Δ χ^2^_(8)_ = 2089.82, *p* < 0.05], indicating that strong factorial invariance across school classes does not hold. Constraining the residual variance at Level 3 to be zero (and freely estimate Level 2 residual variance) did not lead to a significant deterioration of model fit, Δ χ^2^_(8)_ = 6.50, *p* = 0.59. This indicates that strong factorial invariance across schools holds. The M*plus* syntax for the final model can be found in Appendix B.

**Table 1:** **Fit measures of the three-level models**.

**Model**	***df***	**χ^**2**^**	**RMSEA**	**CFI**
1. Baseline model (equal factor loadings across levels)	71	731.95	0.045	0.96
2. Strong factorial invariance at Level 2	79	2821.77	0.088	0.84
3. Strong factorial invariance at Level 3	79	738.45	0.043	0.96

**Figure 1:**
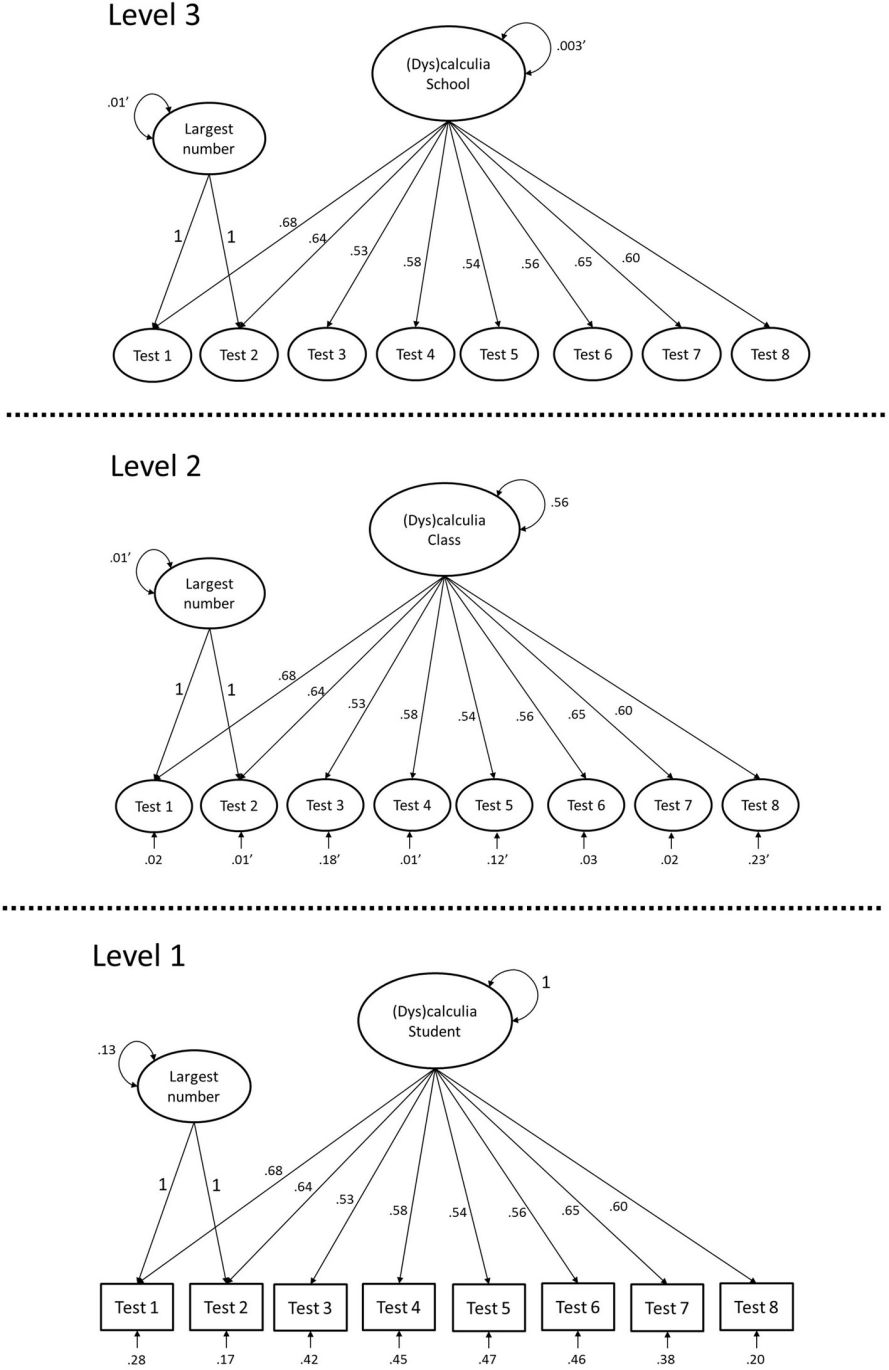
**A three-level factor model with equal factor loadings across levels and no residual variance at Level 3. Parameter estimates are unstandardized. Non-significance is indicated by an apostrophe (′)**.

Figure 1 shows the final model (Model 3) with unstandardized parameter estimates. By inspecting the significance of the residual variance for each indicator at Level 2, it appears that there is significant measurement bias across school classes for Test 1, Test 6, and Test 7. Using the parameter estimates, it can be calculated how much of the variance in these indicators is caused by class level variables other than (dys)calculia. The proportion of residual variance with respect to the total Level 2 variance is calculated as: Residual variance at Level 2/Total variance at Level 2. For Test 1 for example, the total variance at Level 2 is: 0.01 + 0.68^2^ × 0.56 + 0.02 = 0.28, and the Residual variance at Level 2 is 0.02, so the proportion would be 0.02/0.28 = 0.071. The proportion of residual variance with respect to the total variance is calculated as: Residual variance at Level 2/Total variance at Level 1 + Level 2 + Level 3. Table 2 gives an overview of these proportions for the three biased tests. Test 6 shows the most bias, followed by Test 7 and Test 1. However, the proportions of bias can be considered quite small in all tests.

**Table 2:** **Proportions of variance caused by biasing variables at Level 2**.

**Test**	**Proportion bias Level 2**	**Proportion bias Total**
Test 1	0.071	0.019
Test 6	0.146	0.031
Test 7	0.090	0.020

Equality of factor loadings brings the factors on the same scale across levels, which means that the ICC of the factor can be calculated (Mehta and Neale, 2005; Kim et al., 2012). The ICC at Level 2 is equal to 0.56/ (1 + 0.56 + 0.003) = 0.358, indicating that 35.8% of the variance in dyscalculia is at the school class level. At the school level, the ICC is 0.003/ (1 + 0.56 + 0.003) = 0.002, so only 0.2% of the variance in dyscalculia is at the school level.

### Conclusion

The analyses indicated that the screening instrument for dyscalculia cannot be considered fully measurement invariant across school classes. That is, in three of the eight subtests, differences across school classes cannot be fully attributed to differences in the average level of dyscalculia in the school classes. An explanation for the measurement bias can be found by looking at the content of the tests, and trying to distil the class level biasing factor. This is seldom easy, especially if the bias is small. In the current example, an explanation for class level bias in general could be the quality of the instruction that the teachers gave to the children. This is supported by the fact that Test 1 and Test 2 are quite similar (crossing out the largest number) and Test 7 and Test 8 are quite similar (subtraction and addition), but measurement bias across school classes is only found for the first tests of these pairs. In the second tests of each pair, the children already practiced with the type of assignment, rendering quality of the instruction less influential. Test number 6 is about filling in a number on a line, which can be viewed as a different from the other tests in that it forces respondents to visualize numbers on a straight line, which may not match the way students learn mathematics from their teacher. These is no cluster bias detected at the school level. As the number of schools, as well as the number of classes per school in this dataset are very small, a possible explanation of this non-finding is that the test for cluster bias did not have much power to detect bias at the school level.

## Discussion

In this study I illustrated how strong factorial invariance across the Level 2 and Level 3 clustering variable can be investigated. The employed method is only suitable to test strong factorial invariance, by rejecting models with zero residual variance at Level 2 or Level 3. However, the test cannot differentiate between violations of weak and strong factorial invariance. If **Θ**_LEVEL2_ ≠ **0**, this can also be caused by a difference in factor loadings across school classes, which is a violation of weak factorial invariance (Jak et al., 2013). So, if non-zero residual variance is detected, we know that strong factorial invariance does not hold, but we do not know if weak factorial invariance holds. An advantage of the current method is that factorial invariance with respect to Level 2 and Level 3 variables can be tested, even without having measured these variables. Non-zero residual variance at a level indicates bias with respect to some variable at that level, and can thus be viewed as a global test of measurement invariance with respect to any variable. If bias with respect to the clustering variable is found, covariates could be added to the model to explain the bias (Verhagen and Fox, 2012; Jak et al., 2014). In the current dataset this was not possible, as we did not have a measure of the supposed biasing factor, and other covariates at Level 2 did not have significant variance.

### The interpretation of residual variance in multilevel models

With equal factor loadings across levels, at the higher levels of a multilevel factor model, non-zero residual variance always represents measurement bias. This is not the case in single level data (or at Level 1), as we cannot distinguish variance caused by item specific factors from random measurement error variance.

In a factor model, residual variance stems from a residual factor (δ) that consists of two components, a structural component, s, and a random component, e (Bollen, 1998). With VAR() denoting variance:

(6) VAR(δ)=VAR(s)+VAR(e),

in which s represents a specific component, that is unique to the indicator, causing systematic variance in the test score. The remaining part of the residual variance is caused by a random component, e, representing measurement error. The expected value, denoted E(), of the structural component s may be non-zero, and could be interpreted as the intercept in a factor model:

(7) E(s)=τ.

The random component is unsystematic and has an expected value of zero:

(8) E(e)=0.

The residual variance of each indicator is thus equal to the sum of the variance of the two components, and the mean of the residual factor is equal to the mean of the structural component.

Zero structural residual variance represents invariance of the indicator with respect to all variables. As mentioned, in a single level model we cannot distinguish structural residual variance from measurement error variance, rendering it impossible to identify non-zero residual variance as measurement bias. At the second (and higher) level of a multilevel model, it *is* possible to test whether structural variance is present. Given that the cluster mean of the random component is expected to be zero (Equation 8), all residual variance at aggregated levels represents structural variance. Of course, if the number of observations per cluster is very small, some random error variance may be aggregated to the higher level.

### Alternative approaches with two-level data

The test for cluster bias is a useful addition to the existing set of structural equation modeling tools to investigate measurement bias. However, it is not the only test that can be used to investigate measurement invariance across clusters in multilevel data. One of the alternatives to is to test for measurement bias in a fixed effects model, i.e., in a multigroup model in which each cluster is a group. The equal factor loadings and intercepts across groups (clusters) in a multigroup model represent absence of cluster bias. Although this approach is possible in principle, it is hardly practical when the number of clusters is large. Muthén and Asparouhov (2013) describe an alternative way to circumvent the cumbersome strategy of multigroup modeling with large numbers of groups, using a 2-step procedure with Bayesian estimation. They introduce the concept of “approximate measurement invariance,” referring to the analysis of measurement invariance across several groups using Bayesian SEM (BSEM), see also Van De Schoot et al. (2013). In Step 1 of the procedure (the analysis of approximate measurement invariance), in each group the difference between the group specific measurement parameter (factor loading or intercept) and the average of the particular parameter across all groups is estimated. The researcher can then identify the group with the largest difference between its measurement parameter and the average parameter as the most deviant group. In the next step, using BSEM, one estimates a model in which all factor loadings and intercepts are equal across groups, except for the groups that were identified as deviant in the previous step. This is similar to the use of modification indices with maximum likelihood estimation in a multigroup model, where the most deviant group will show the largest modification index in an analysis with equal factor loadings and intercepts. An advantage of the BSEM method is that it works well for the analysis of categorical variables, while maximum-likelihood estimation with categorical variables often leads to computational problems due to the numerical integration involved. A disadvantage of the approximate measurement invariance approach is that it relies on prior distributions for the model parameters, and different priors may yield different outcomes. Muthén and Asparouhov recommend zero-mean, small-variance priors for the difference parameters. However, the optimal size of the small-variance of the priors is a subject of debate. When trying to analyse the dyscalculia data using the BSEM method, it was unsuccessful due to the enormous computational load with 156 groups. Indeed, I have not seen applications of the BSEM method with large numbers of groups.

A framework for the detection of measurement bias across large numbers of groups within Bayesian Item Response Theory (IRT) is given by Verhagen and Fox (2012), using multilevel random item effects models (De Jong et al., 2007; Fox and Verhagen, 2010). Verhagen and Fox estimate a random effects parameter for all measurement parameters in the model (i.e., discrimination parameters and difficulty parameters in an IRT model), and test which of the measurement parameters have significant variance across clusters using Bayes factors or using the Deviance Information Criterion (DIC). Consequently, the cluster level variance in item parameters may be explained by adding covariates to the model. The approach of Verhagen en Fox is similar to the approach in this article in some respects. Both approaches treat groups as randomly drawn from a population of groups. Both approaches test the hypothesis of zero variance of parameters at the cluster level, and both allow for the explanation of non-zero variance by cluster level variables. The main differences between the two approaches relate to the modeling framework (multilevel IRT vs. multilevel SEM), and the estimation method [Bayesian estimation vs. frequentist (maximum likelihood) estimation]. It is an interesting topic of future research to compare the outcomes of the two methods.

### Alternative approaches with three-level data

Although it seems straightforward to analyse three-level data with the before mentioned approaches as well, I am not aware of any published articles in which measurement invariance with respect to the Level 2 and Level 3 cluster variables is investigated. One option would be to treat the Level 3 clustering as fixed, and impose the measurement invariance restrictions on the two-level models for every school. That is, first measurement invariance across school classes can be investigated using the test for cluster bias (Jak et al., 2013) for each school separately, and next the equality of factor loadings and intercepts can be tested across schools (see Muthén et al., 1997). This approach is not considered very useful, as within each school, the number of school classes will never be large enough to obtain stable estimates and have acceptable power to reject measurement invariance. The BSEM approach can probably be extended to three-level data, by including difference parameters for the intercepts and factor loadings at the school level as well as at the class level. One difference parameter would then reflect how the specific school average differs from the overall average, and another difference parameter would reflect how the specific class deviation from the school average differs from the average class deviation from the school average. The method of Verhagen and Fox could also be extended to three-level data, by estimating school level variance for each measurement parameter.

Although the three-level SEM method is not the only option to investigate measurement bias in three-level data, it is shown in this article that it is at least a relatively simple method to use. At the higher levels of multilevel data, the power of the statistical tests may not be very large, as the number of higher level units is often small. In the current example there were 50 schools at Level 3. From simulation research with two-level data (Jak and Oort, under review), we know that with 50 clusters of size 5, the power to detect large bias is only 50%. Extrapolating this to the three-level situation indicates that that in our example, we did not have high power to detect bias at Level 3. Nevertheless, the illustration can be useful as an example of how the detection of measurement invariance in three-level data may be executed.

### Conflict of interest statement

The author declares that the research was conducted in the absence of any commercial or financial relationships that could be construed as a potential conflict of interest.

## References

[B1] Bollen K. A. (1998). Structural Equation Models. New York, NY: John Wiley & Sons, Ltd

[B2] Browne M. W. Cudeck R. (1992). Alternative ways of assessing model fit. Sociol. Methods Res. 21, 230–258 10.1177/0049124192021002005

[B3] Butterworth B. (2005). Developmental dyscalculia, in The Handbook of Mathematical Cognition, ed Campbell J. D. (New York, NY: Psychology Press). 455–469

[B4] De Jong M. G. Steenkamp J. B. E. Fox J. P. (2007). Relaxing measurement invariance in cross−national consumer research using a hierarchical IRT model. J. Consum. Res. 34, 260–278 10.1086/518532

[B5] Devine A. Soltész F. Nobes A. Goswami U. Szûcs D. (2013). Gender differences in developmental dyscalculia depend on diagnostic criteria. Learn. Instr. 27, 31–39 10.1016/j.learninstruc.2013.02.004 PMC4461157 27667904

[B6] Fox J. P. (2010). Bayesian Item Response Modeling: Theory and applications. New York, NY: Springer 10.1007/978-1-4419-0742-4

[B7] Fox J.-P. Verhagen A. J. (2010). Random item effects modeling for cross-national survey data, in Cross-Cultural Analysis: Methods and Applications, eds Davidov E. Schmidt P. Billiet J. (London: Routledge Academic), 467–488

[B8] Gross-Tsur V. Manor O. Shalev R. S. (1996). Developmental dyscalculia: prevalence and demographic features. Dev. Med. Child Neurol. 38, 25–33 10.1111/j.1469-8749.1996.tb15029.x 8606013

[B10] Hu L. Bentler P. M. (1999). Cutoff criteria for fit indices in covariance structure analysis: conventional versus new alternatives. Struct. Equ. Modeling 6, 1–55 10.1080/10705519909540118

[B12] Jak S. Oort F. J. Dolan C. V. (2013). A test for cluster bias: detecting violations of measurement invariance across clusters in multilevel data. Struct. Equ. Modeling 20, 265–282 10.1080/10705511.2013.769392

[B13] Jak S. Oort F. J. Dolan C. V. (2014). Measurement bias in multilevel data. Struct. Equ. Modeling Multidiscip. J. 21, 31–39 10.1080/10705511.2014.856694

[B14] Kim E. S. Kwok O. M. Yoon M. (2012). Testing factorial invariance in multilevel data: a Monte Carlo study. Struct. Equ. Modeling Multidiscip. J. 19, 250–267 10.1080/10705511.2012.659623

[B15] Kosc L. (1974). Developmental dyscalculia. J. Learn. Disabil. 7, 164–177 10.1177/002221947400700309

[B17] Mehta P. D. Neale M. C. (2005). People are variables too: multilevel structural equations modeling. Psychol. Methods 10:259 10.1037/1082-989X.10.3.259 16221028

[B18] Mellenbergh G. J. (1989). Item bias and item response theory. Int. J. Educ. Stat. 13, 127–143

[B19] Mellenbergh G. J. (1994). A unidimensional latent trait model for continuous item responses. Multivariate Behav. Res. 29, 223–236 10.1207/s15327906mbr2903_2 26765136

[B20] Meredith W. (1993). Measurement invariance, factor analysis, and factorial invariance. Psychometrika 58, 525–543 10.1007/BF02294825

[B21] Milikowski M. Vermeire S. (2013). Nederlandse Dyscalculie Screener (NDS). Handleiding en Verantwoording, Amsterdam: Boom Testuitgevers

[B22] Millsap R. E. Everson H. (1991). Confirmatory measurement model comparison using latent means. Multivariate Behav. Res. 26, 479–497 10.1207/s15327906mbr2603_6 26776714

[B23] Muthén B. (1990). Mean and Covariance Structure Analysis of Hierarchical Data. Los Angeles, CA: UCLA statistics series, NO 62

[B24] Muthén B. Asparouhov T. (2013). New Methods for the Study of Measurement Invariance with Many Groups. Technical report. Available online at: http://www.statmodel.com

[B25] Muthén B. O. Khoo S. T. Gustafsson J. E. (1997). Multilevel Latent Variable Modeling in Multiple Populations. Technical report. Available online at: http://www.statmodel.com

[B26] Muthén L. K. Muthén B. O. (1998–2012). Mplus User's Guide. 7th Edn. Los Angeles, CA: Muthén and Muthén

[B27] Rabe-Hesketh S. Skrondal A. Pickles A. (2004). Generalized multilevel structural equation modelling. Psychometrika 69, 167–190 10.1007/BF02295939

[B29] Stoel R. D. Garre F. G. Dolan C. V. van den Wittenboer G. (2006). On the likelihood ratio test in structural equation modeling when parameters are subject to boundary constraints. Psychol. Methods 11, 439–455 10.1037/1082-989X.11.4.439 17154756

[B30] Vandenberg R. J. Lance C. E. (2000). A review and synthesis of the measurement invariance literature: suggestions, practices, and recommendations for organizational research. Organ. Res. Methods 2, 4–69 10.1177/109442810031002

[B31] Van De Schoot R. Kluytmans A. Tummers L. Lugtig P. Hox J. Muthen B. (2013). Facing off with Scylla and Charybdis: a comparison of scalar, partial, and the novel possibility of approximate measurement invariance. Front. Psychol. 4:770 10.3389/fpsyg.2013.00770 PMC3806288 24167495

[B32] Verhagen A. J. Fox J.-P. (2012). Bayesian tests of measurement invariance. Br. J. Math. Stat. Psychol. 66, 383–401 10.1111/j.2044-8317.2012.02059.x 23039871

[B33] Wicherts J. M. Dolan C. V. (2010). Measurement invariance in confirmatory factor analysis: an illustration using IQ test performance of minorities. Educ. Meas. Issues Pract. 29, 39–47 10.1111/j.1745-3992.2010.00182.x

[B34] Widaman K. F. Reise S. P. (1997). Exploring the measurement invariance of psychological instruments: applications in the substance use domain, in The Science of Prevention: Methodological Advances from Alcohol and Substance Abuse Research, eds Bryant K. J. Windle M. West S. G. (Washington, DC: American Psychological Association), 281–324 10.1037/10222-009

