# Supplementary material for: Testing strong factorial invariance using three-level structural equation modeling
Source: Front Psychol. 2014 Jul 25;5:745. doi: 10.3389/fpsyg.2014.00745 (PMC4110441; doi:10.3389/fpsyg.2014.00745)
Supplement: Supplementary file 1 [file Presentation1.PDF]

Appendix A. The eight subtests of the screening instrument

Test 1 - Largest number

The goal is to cross-out the largest number of two numbers that are smaller than 10, for example:

~~2~~ or 1

6 or ~~8~~

Test 2 - Largest number

Cross out the largest number of sets with numbers larger than 10, for example:

46 or ~~64~~

29 or ~~51~~

Test 3 - ***From dots to numbers***

Count the number of dots (the dots differ in their size).

•●•● (4)

Test 4 - Number series

Fill in the number between two other numbers, for example:

20 _ 22 (fill in 21)

20 _ 18 (fill in 19)

Test 5- Even or odd

Cross out the even numbers, for example:

3 7 9 5 3 7 9 ~~8~~ ~~6~~ 3

Test 6 - Number line until 10

Which number would be on this position?

1__________-______10 (fill in 6)

Test 7 - Fast subtraction

Subtract a number from a given number, for example:

Subtract 2 from:

4 8 6 3 9

Test 8 - Ten or more

Rows of addition questions are given. The goal is the cross out the items with an outcome larger than 10.
