# Supplementary material for: Testing strong factorial invariance using three-level structural equation modeling
Source: Front Psychol. 2014 Jul 25;5:745. doi: 10.3389/fpsyg.2014.00745 (PMC4110441; doi:10.3389/fpsyg.2014.00745)
Supplement: Supplementary file 2 [file Presentation2.PDF]

Appendix B. M*plus* input for the final model (Model 3)

```
Title:      Model 3
Data:       file is dyscalculia.dat;
Variable:   names are
            schoolcluster classcluster vr0 - vr8;
            usevariables are v1 - v8;
            cluster is schoolcluster classcluster ;

Define:     v1 = vr1/12;      !divided by the SD’s (roughly) to
            v2 = vr2/10;      !equalize the scale of the variables
            v3 = vr3/10;
            v4 = vr4/7;
            v5 = vr5/25;
            v6 = vr6/9;
            v7 = vr7/9;
            v8 = vr8/17;

Analysis:   TYPE = THREELEVEL;
            estimator = MLF;

Model:     %WITHIN%
           calc by v1* v2 - v8 (Lambda1-Lambda8);  ! labels
           calc@1;                  ! L1 factor variance at 1

           number by v1@1 v2@1;     ! both factor loadings fixed at 1
           number;                  ! variance = residual covariance

           calc with number@0;      ! factors are uncorrelated

           V1-v8;       ! free residual variance


           %BETWEEN classcluster%
           L2calc by v1* v2 - v8 (Lambda1-Lambda8);   ! same labels
           L2calc;                  ! L2 factor variance free

           L2number by v1@1 v2@1;   ! both factor loadings fixed at 1
           L2number;                ! variance = residual covariance

           L2calc with L2number@0;  ! factors are uncorrelated

           V1-v8;       ! free residual variance (measurement bias)


           %BETWEEN schoolcluster%
           L3calc by v1* v2 - v8 (Lambda1-Lambda8);   ! same labels
           L3calc;                  ! L3 factor variance free

           L3number by v1@1 v2@1;   ! both factor loadings fixed at 1
           L3number;                ! variance = residual covariance

           L3calc with L3number@0;

           v1-v8@0;     ! residual variance at 0 (no measurement bias)
```
